# Influence of Body Composition on Physical Fitness in Adolescents

**DOI:** 10.3390/medicina56070328

**Published:** 2020-07-02

**Authors:** María Mendoza-Muñoz, José Carmelo Adsuar, Jorge Pérez-Gómez, Laura Muñoz-Bermejo, Miguel Ángel Garcia-Gordillo, Jorge Carlos-Vivas

**Affiliations:** 1 Health, Economy, Motricity and Education (HEME) Research Group, Faculty of Sport Sciences, University of Extremadura, 10003 Cáceres, Spain; mamendozam@unex.es (M.M.-M.); jorge.carlosvivas@gmail.com (J.C.-V.); 2 Social Impact and Innovation in Health (InHEALTH), University of Extremadura, 10003 Cáceres, Spain; lauramunoz@unex.es; 3 Facultad de Administración y Negocios, Universidad Autónoma de Chile, sede Talca 3467987, Chile; miguelgarciagordillo@gmail.com

**Keywords:** fitness, health markers, obese, obesity, prevention, secondary school, youth

## Abstract

*Background and objectives:* Childhood obesity is one of the main public health issues facing advanced societies. The Spanish population presents 28.6% overweight or obese adolescents, and Extremadura, 22.3%. Physical fitness is considered one of the most important health markers and a common predictor of morbidity and mortality caused by cardiovascular diseases. Thus, fitness tests are needed for health monitoring, especially in overweight and obese adolescents. Therefore, this study aimed to explore the relationship between body composition and physical fitness in adolescents and to analyse if there are differences in physical condition and body composition between the sexes, as well as to compare the different body mass index (BMI) categories to each other. This project also tried to find regression equations to predict the physical fitness test results. *Materials and Methods:* A total of 225 adolescents were recruited. Weight status was classified according to WHO 2007 standards. Body composition variables (i.e., BMI, fat mass (FM), FM percentage (FM%), and fat-free mass (FFM)) and physical fitness (standing long jump (SLJ), speed–agility (SA), cardiorespiratory fitness (CF), and handgrip strength (HS)) were assessed. *Results:* Significant differences were found between the sexes in body composition (FM%, FM, and FFM) and physical fitness (SLJ, SA, CF, and HS) (*p* < 0.001) in favour of males. Significant differences were also found in speed (*p* = 0.002), CF (*p* < 0.001), and SLJ (*p* = 0.004) in favour of normal-weight adolescents compared to overweight and obese adolescents. Contrarily, the outcomes revealed a significantly greater HS (*p* = 0.014) in favour of overweight and obese participants compared to normal-weight adolescents. Moreover, the results showed that CF and SLJ correlated inversely with BMI, FM%, and total FM. There was also a direct relationship between SA and FM percentage, as well as between HS and FFM. Finally, four fitness test predictive models are proposed based on body composition, age, sex, and BMI. *Conclusions:* Overweight and obese adolescents have lower levels of physical fitness than their normal-weight peers, regardless of their sex. Normal-ranged body composition values are related to a greater physical fitness in adolescents. Furthermore, this study presents several equations that can help to predict the performance of different fitness tests in adolescents.

## 1. Introduction

Childhood obesity is one of the main public health issues that should be attended to in advanced societies [1]. Several studies have aimed to determine the obesity and overweightness levels in children and youths in Spain, because of their high incidence [2,3,4,5,6]. The Spanish National Health Survey (SNHS) [7], which assessed children and adolescents aged between 2 and 17 years, concluded that the percentage of overweight or obese children and adolescents in Spain was 28.6% in 2017. This prevalence was similar in boys and girls both for overweight (18.30% vs. 18.20%) and obese (10.40% vs. 10.2%) adolescents, respectively. Specifically, this study [7] revealed that the percentage of overweightness and obesity in Extremadura was 11.9% and 10.4%, respectively. However, the percentage or total sample size proceeding from the region of Extremadura is not specified.

Likewise, the last national-level study on this topic, named the PASOS study, showed 20.7% and 14.2% overweightness and obesity, respectively, in children and adolescents aged between 8 and 16 years. However, this study neither indicated the sample size of every region nor differentiated between sex. Moreover, all centres recruited from Extremadura in this study were located in the province of Badajoz. Thus, there are no representative data from the province of Cáceres despite this area practically including the other half of the total population of Extremadura [4]. 

Both the PASOS and SNHS studies show the high prevalence of child and youth obesity and overweightness in Extremadura (PASOS: 34.9% vs. SNHS: 22.6%). Due to the lack of specificity, the need for continuous updating, and the scarcity of epidemiological data on overweightness, obesity, and physical fitness, an observatory named the Well-Being, Obesity, and Motricity Observatory in Childhood and Youth (WOMO) was created, to monitor the weight and fitness status of children and adolescents, knowing their lifestyles, and developing or proposing different strategies to combat and prevent this problem. Specifically, it was created to evaluate the 10- to 17-year-old population due to the lack of data [8].

One of the most important factors to prevent overweightness and obesity in child, adolescent, and adult populations is physical activity [9]. Regarding physical fitness, several studies have determined the benefits of health-related physical fitness in children and youths [9,10,11]. Physical fitness is considered one of the most important health markers and is commonly understood as a predictor of morbidity and mortality for cardiovascular diseases [11,12]. More specifically, low muscular fitness is associated with mortality in adulthood [12], stabilising a relationship between low muscular fitness with cardiovascular disease risk factors (CVD) [11] and metabolic syndrome in young people [13,14]. It has also been shown that adolescents with low levels of muscular fitness present an increased risk of maintaining low muscular fitness levels in their adulthood [15]. In addition, greater isometric muscle strength during the adolescence has been associated with lower levels of CVD during young adulthood independently of fitness, adiposity, and other confounding factors [16]. Thus, fitness tests are needed for health monitoring, especially in overweight and obese adolescents, who generally show a poorer physical condition than their normal-weight peers [17,18,19,20,21].

Furthermore, we must highlight the importance of sex differentiation. Adolescence is a time of multiple physical and emotional changes, which affect the physical condition. Several studies have affirmed that strength levels, speed/agility, performance, and aerobic capacity are higher in males than females [22,23,24]. Due to these differences, we can find that the normative values of physical condition are established according to sex [23]. Thus, the differences between boys and girls should be considered when designing physical activity programs.

Previous studies have explored the influence of sex, age, and body composition on fitness status [25,26,27,28]. Thus, predictive equations have been previously used in children to estimate basic motor abilities (e.g., balance, speed, agility, power, coordination, and reaction time) and health-related fitness items (e.g., flexibility, muscle strength and endurance, and cardiovascular functions) [27], fitness tasks (e.g., grip strength, step test, sit and reach, and timed sit-ups) [25], or make predictions of physical condition (e.g., 30-m dash, flamingo balance, seated chest pass, standing long jump (SLJ)) in non-obese children [26] based on age, sex, anthropometry, or body composition. Few studies have proposed predictive equations based on sex, age, and BMI to estimate physical condition in adolescents [29,30]. However, to our knowledge, no studies have considered body composition parameters when developing these fitness predictive equations.

Therefore, this study aimed to explore the physical condition and body composition of adolescents and the possible differences between the sexes and BMI categories. This project also aimed to analyse the relationship between body composition and physical condition in this population. Moreover, this project attempts to find a regression equation to predict the physical fitness test result based on body composition, BMI, age, and sex. 

## 2. Materials and Methods

### 2.1. Study Design

A single-measure cross-sectional study was conducted.

### 2.2. Ethics Approval

The study protocol and procedures were conducted according to the Declaration of Helsinki and approved by the Bioethics and Biosafety Committee at the University of Extremadura (approval number: 138-2019; 155-2019; 139-2019).

### 2.3. Sample Size

A sample size of 147 achieves 90% power to detect a difference of −0.20 between the null hypothesis correlation of 0.40 and the alternative hypothesis correlation of 0.60 using a two-sided hypothesis test with a significance level of *p* ≤ 0.05.

### 2.4. Participants 

Four secondary schools from Extremadura were contacted to participate in this study. A total of 225 adolescents (108 males and 117 females), aged between 12 and 17 years old were recruited. Participants needed to meet the following eligibility criteria to be included in this project: (1) age: 12–17 years old; (2) registered and/or living in the autonomous community of Extremadura; (3) not presenting pathologies that prevent participation in physical fitness tests, physical activity, or require special attention; (4) authorised by parents or legal guardians; (5) adolescents’ acceptance to participate in the study.

### 2.5. Procedures and Measures

In this project, a variety of tools were used to assess physical condition and body composition. We followed the procedures proposed in the study protocol WOMO [8], which allowed controlled outcomes associated with monitoring overweightness and obesity, as well as the lifestyles of children and adolescents.

Before the first measurement, all participants carried out a familiarisation phase, which consisted of an explanation and practice trial of each of the included tests and evaluations. 

**Anthropometric measurements**. These were taken under standardized conditions, and following the proposal of the World Health Organization (WHO) for the development of the Childhood Obesity Surveillance Initiative (COSI) [2] and ALADINO report [3]. The protocol set out in the Data Collection Procedure Manual developed specifically for the COSI initiative [2] was followed at all times. The height was measured with a height rod (TANITA Tantois, Tanita Corporation, Tokyo, Japan). The instrument was placed on a vertical surface so that the measurement scale was perpendicular to the ground. The height was measured standing up, with shoulders balanced and arms relaxed along the body. The measurement was taken in cm, up to the nearest mm. Bodyweight was measured using a bioelectrical impedance analyser (BIA) (Tanita MC-780 MA, Tanita Corporation, Tokyo, Japan). The evaluation was carried out in the “standard mode”, introducing the participant’s age, sex, and height. Bodyweight was recorded in kg, up to the nearest 100 g. The BIA was also used to evaluate fat mass (FM) and fat-free mass (FFM). In addition, all recommendations for proper BIA use were followed [31,32]: using more than 3 h after getting up, urinating before the measure, no food and drink for the last 3 h, no excess food and drink the day before, no heavy exercise for the last 12 h, no alcohol for the last 12 h, no metal objects, or people with pacemakers. The BIA technology, compared to dual X-ray absorptiometry (DXA) used in some studies [33], offers a great reproducibility when assessing obese adolescents’ FM (ICC = 0.88), FM% (ICC = 0.66), or FFM (ICC = 0.76). Additionally, DXA and BIA have shown high correlations in FM% (r = 0.852, ICC = 0.84, and concordance coefficient = 0.844) and FFM (r = 0.976, ICC = 0.95, and concordance coefficient: 0.955) measurements in youths regardless of their physical activity level [34]. 

**Physical condition.** The Assessing Levels of Physical Activity fitness test battery *(ALPHA-fitness test battery*) was applied [35,36]. The following assessments were included: (1) upper body strength (hand dynamometry); (2) lower limb strength (standing long jump (SLJ)); (3) cardiorespiratory fitness (20-m shuttle run test); and (4) speed and agility (4 × 10 m test).

1. *Handgrip strength (HS)*. Maximum HS was measured by maximum hand dynamometry [37,38,39] using a digital dynamometer with an adjustable grip (TKK 5041 Grip D, Takei, Tokyo, Japan). A table of estimated reference values was used to adjust the grip [35].
2. *Lower limb strength*. Feet together SLJ was used. Participants performed two attempts. The trial with the longest distance was considered for analysis. It was measured in cm using a PVC measuring tape (from the take-off line to the point where the back of the heel nearest to the take-off line landed on the ground) [40].
3. *Cardiorespiratory fitness (CF)* was assessed using the 20-m shuttle run test. Participants ran between two lines separated by 20 m. Speed control was performed using an audio signal previously established. The test started at 8.5 km/h, and the speed increased by 0.5 km/h every minute. Participants started at the first audio signal or beep. The test finished when the participant stops due to fatigue or fails to reach the end line concurrent with the audio signal or beep on two consecutive occasions [41,42]. The last half stage completed was considered for analysis.
4. *Speed and agility (SA)* was measured by the 4 × 10 m test [36]. Participants ran a distance back and forth between two lines 10 m apart taking three sponges alternately, as quickly as possible (covering a total distance of 40 m).

**Overweightness and Obesity.** Obesity was assessed through BMI following WHO methodology [2,43]. BMI was calculated using the formula: bodyweight (kg) divided by height squared (m^2^). The weight status (low weight, normal weight, and overweight and obese) was established based on the standard deviation (SD) criteria [43].

### 2.6. Statistical Analysis

All collected information was tabulated in a database specifically designed for this purpose. Statistical analyses were carried out using the software IBM SPSS Statistics 25 (data were de-identified). Data are presented as means (SD) and medians (interquartile range) both for the total sample and the three different categories where participants were divided: (1) males vs. females; (2) low weight vs. normal weight vs. overweight and vs. obese; and (3) overweight and obese males vs. overweight and obese females. The COSI initiative criteria [2] and the WHO growth standards [43] were used to divide the sample in “low-weight”, “normal-weight”, “overweight”, and “obese” adolescents. Thus, every adolescent’s weight status was established based on the SD criteria: (1) “low-weight” BMI < −3 SD; (2) “normal-weight” BMI < −2 SD; (3) “overweight” BMI > +1 SD; and (4) “obese” BMI > +2 SD. “Overweight” and “obese” categories were considered as a single group when performing the study computations and comparisons. Normality and homogeneity were checked using the Kolmogorov–Smirnov and Levene’s tests, respectively. Between-group differences were analysed using independent Student’s *t*-tests (normally distributed variables) and Mann–Whitney U tests (non-normally distributed variables). Significant differences were considered for *p* ≤ 0.05. The relationships between the different dependent variables was quantified applying Pearson’s (parametric) and Spearman’s (non-parametric) correlation coefficients. Then, a Bonferroni correction for multiple comparisons was applied, setting the alpha significance level at 0.007. Correlation thresholds were established following Cohen’s classification [44,45]: 0.30–0.59 moderate; 0.6–0.79 high, and ≥ 0.8 excellent. Specific regressions of fitness tests on BMI, FM percentage, age, and sex were also done. Every fitness test was considered as a dependent variable, and BMI, FM%, age, and sex were the independent variables. The obtained equations can be used to predict the performance in every fitness test. A significance level lower than 0.001 was required to introduce a new variable into each prediction model. The overall predictive power was evaluated by adjusted R^2^.

## 3. Results

### 3.1. Study Population Characteristics

The total sample of this study included 52% females and 48% males.

Table 1 shows the participants’ anthropometry and physical fitness test outcomes stratified by sex. Females showed higher FM% (*p* < 0.001) and FM (*p* < 0.001) compared to males. In contrast, males showed significantly higher FFM (*p* < 0.001) than females. Likewise, the fitness test outcomes revealed a significantly higher performance of boys on SLJ, SA, CF, and HS (*p* < 0.001) compared to girls.

Table 2 shows the anthropometry and physical fitness test outcomes stratified by BMI categories (low weight, normal weight, overweight, and obese). The results revealed that FM%, FM, and FFM significantly differed between the BMI categories of the total sample. 

All fitness tests also showed significant differences comparing normal-weight, overweight, and obese adolescents. Normal-weight adolescents presented a significantly greater performance than their overweight or obese counterparts in SA (*p* = 0.002), CF (*p* < 0.001), and SLJ (*p* = 0.004). However, overweight and obese adolescents showed significant differences in HS (*p* = 0.014) compared to their normal-weight peers.

Table 3 displays obese and overweight adolescents’ anthropometry and physical fitness outcomes stratified by sex. The results revealed significantly higher FFM (*p* < 0.001), SLJ (*p* < 0.001), SA (*p* < 0.001), CF test (*p* < 0.001), and HS performance (*p* < 0.001) in overweight/obese boys compared to girls. Contrarily, there were significantly higher FM% (*p* < 0.001) and FM (*p* = 0.020) in overweight/obese girls compared to boys.

### 3.2. Bivariate Correlations between Variables

Table 4 shows the bivariate relationships between body composition variables and physical fitness tests stratified by sex and BMI. 

The results revealed that CF and SLJ were inversely associated with BMI, FM%, and total FM in adolescents. The most consistent association for all participants, for all boys and lower weight adolescents, was observed between FM markers and CF. A positive association between SA and FM% and between FFM and HS were also found for all BMI categories.

Overall, all BMI categories showed that SA was inversely associated with SLJ, CF, and HS. Additionally, CF directly correlated with SLJ and HS, and HS with SLJ.

### 3.3. Fitness Test Predictive Models

Specific regressions of all fitness tests on BMI, FM%, age, and sex were carried out. Thus, we propose the following equations:

CF = 3.643 + FM% (−0.149) + Age (0.337) + Sex (1.189) (R^2^ = 0.411; *p* < 0.001)

HS = −10.161 + Sex (5.566) + Age (1.522) + BMI (0.643) (R^2^ = 0.332; *p* < 0.001)

SLJ = 168,35 + FM% (−3.480) + BMI (3.303) (R^2^ = 0.356; *p* < 0.001)

SA = 23.639 + FM% (0.70) + Age (−0.373) + Sex (−0.501) (R^2^ = 0.294; *p* < 0.001)

where CF is cardiorespiratory fitness test performance as the last completed 20-m shuttle test stage, HS is handgrip strength in kg, SLJ is standing long jump distance in cm, and SA is the 4 × 10 m test performance in s.

## 4. Discussion

This study explored the relationship between the physical condition and body composition of adolescents, establishing differences in physical condition and body composition variables between the sexes and BMI categories. We also propose several equations that can help to predict or estimate the physical fitness tests results (CF, SA, HS, and SLJ) based on BMI, FM%, age, and sex.

Several studies have assessed the level of overweightness and obesity in children and adolescents [2,3,4,5,6], as well as evaluated their physical condition compared to their normal-weight peers [17,18,19,20,21]. Furthermore, previous studies have reported between-sex differences in physical fitness and body composition [22,23,24,46]. Despite that, to our knowledge, our study is the first that shows specific data of overweightness, obesity, and fitness condition in adolescents of the Extremadura region, considering its two provinces.

Similarly to previous studies [17,18,19,20,21], our study showed that overweight and obese adolescents had poorer performance in CF, SA, and SLJ than their normal-weight peers. Furthermore, our between-sex comparisons results revealed the greater physical fitness of males compared to females. These findings agree with previous studies that have reported a superior fitness test performance of males adolescents in comparison with their female counterparts [22,23,24]. In contrast, another study did not find a significative difference between the sexes in fitness tests [46]. This difference could be explained by the higher homogeneity of the sample in the study by Ortega et al. [46]. Moreover, our results also showed that overweight and obese boys had a significantly greater performance in SLJ, CF, SA, and HS compared to girls. 

In accordance with previous studies [47,48], our study showed that overweight and obese youth present lower CF compared to their normal-weight counterparts. It could be due to excess FM reducing an individual’s exercise tolerance and aerobic capacity [47]. It has been demonstrated that moderate or high levels of CF are associated with a lower abdominal adiposity in both boys and girls [49]. Overweight and obese adolescents also showed poorer SLJ compared to their normal-weight peers. Previous studies reported similar results, showing that a higher bodyweight could be a barrier in fitness tests where quick position changes are required since bodyweight increased the exerted forces during knee extension [50,51]. Moreover, a significantly greater performance in SA was found in normal-weight adolescents compared to overweight and obese adolescents (*p* = 0.002). These differences are in agreement with other studies that also observed poorer performances of overweight or obese individuals in different fitness tests. These outcomes could be explained by the need for a higher propulsion or lifting of the body mass required by obese adolescents compared with their non-obese peers [17,18], since obese people present an excess FM, which supposes an extra load to move during dynamic activities, and thus, fitness tests. 

Nevertheless, we found overweight and obese adolescents showed greater HS, especially in boys. Previous studies analysed both static and explosive strength and compared lean and obese adolescents [20,52]. They concluded that obese adolescents obtained a better performance during static exercises than normal-weight adolescents. However, opposite results were reported for explosive or dynamic tests, where normal-weight individuals have a greater performance [33,50]. The higher strength levels may be explained by a higher amount of FFM, since underweight adolescents present a lower FFM than obese youths in absolute terms, as has been reported by previous studies [51,53,54]. 

The present study also analysed the relationship between body composition and fitness condition in adolescents. The main outcomes showed that both CF and SLJ are inversely associated with FM% and total FM. We found a stronger association between FM% and these fitness variables in normal-weight adolescents (SLJ: r = −0.571, SA: r = 0.268, CF: r = −0.604, HS: r = −0.373). Similar to previous studies [21,55], the association between SLJ, SA, and CF with FM markers was weaker in female than in male participants. The most consistent association was observed between FM% and 20-m shuttle run test performance for all participants (r > −0.563), boys (r > −0.545), normal-weight (r > −0.604), and obese and overweight adolescents (r > −0.493). These results were consistent independently of the FM markers considered; however, the strongest associations were obtained using the FM%. We also observed a direct association between SA and FM% (r > 0.406), which was greater in obese and overweight adolescents (r > 0.493) compared to normal-weight adolescents (r > 0.268). As we highlighted above, this direct association may be due to the higher FM of overweight and obese adolescents, since FM may be decisive in tests that require rapid mobilization of the body, especially in this population [17,18].

Overall, the obtained results highlight the importance of body composition and its influence on physical fitness levels in adolescents. Thus, this study proposes four different fitness test predictive models, according to youth characteristics that could help to quickly obtain an estimated general vision of adolescents’ health statuses in a short time and knowing a few characteristics of the individuals. The four equations obtained in this study present acceptable R^2^ values (CF: R^2^ = 0.411; HS: R^2^ = 0.322; SLJ: R^2^ = 0.356; SA: R^2^ = 0.294). Thus, these equations could properly predict the results in the different fitness tests previously indicated. Previous studies have reported predictive equations to estimate incremental shuttle walk test performance in adolescents, considering sex, age, and BMI [29,30]. These studies reported an R^2^ score of 54% [29] and 48% [30], respectively. Similarly, Milanese et al. [26] showed a predictive equation for the 30-m dash test, but in children and considering only sex and age. Its R^2^ value (R^2^ = 0.55) was lower than that obtained in our study. There is no doubt that age, sex [25,26,27,29,30], and BMI [25,28] are determining factors for fitness status. Nevertheless, this study [26] suggests that other variables, such as muscle fibre recruitment or muscular coordination, could be considered when developing prediction equations, because they may influence the performance on fitness tests. Moreover, Milanese et al. [26] reported that FFM and FM% were not significant determinants of physical fitness. However, based on our study results, the FM% should be considered, especially for developing dynamic test predictive equations. These differences may be due to the different samples of the studies, since Milanese et al. [26] only included non-obese children, while our study was conducted in adolescents independently of their BMI condition. 

One of the strengths of this study is that physical fitness was assessed using the ALPHA fitness test battery, which is a valid, reliable, applicable, and health-related battery for adolescents [35,40,56]. This study also presents several regression equations, which allows for saving time when measuring physical fitness, facilities or materials for testing are not needed, and the equations do not require specific training for the evaluator. Thus, they are very useful for estimating physical condition in times of lockdown and the use of equations is also cheaper in epidemiological studies and can estimate the results of field tests if they can be done.

Nevertheless, this study has some limitations. It is important to indicate that, although this study based its findings on a representative sample of participants from Extremadura, a larger sample size could improve the strength of the R^2^ values and the associations between the analysed variables. Another limitation is the use of BIA to measure body composition because is not the gold standard instrument to assess it. However, great agreement and concordance have been shown between BIAs an DXA in the targeted population (ICC > 0.99) [33]. Thus, BIA offers an acceptable and reproducible alternative to assess body composition in adolescents [33,34]. Nevertheless, it should be noted that the use of BIA in morbidly obese adolescents is unclear, since the correlation between BIA and DXA decreases as fat mass increases [33]. In this context, it is important to note that none of the participants in this study was morbidly obese, and thus it did not affect our results. 

The obtained results are important to determine the level of overweightness and obesity in adolescents in Extremadura and their values in physical fitness. This evaluation is useful when undertaking the appropriate interventions to improve the results and prevent the high values of overweight and obese adolescents. Therefore, the next step could be an intervention on physical condition to prevent obesity.

## 5. Conclusions

Overweight and obese adolescents have lower levels of physical fitness than their normal-weight peers, regardless of their sex. Normal-ranged body composition values are related to a greater physical fitness in adolescents. Furthermore, this study presents several equations that can help to predict performance on different fitness tests. These equations would be useful for estimating physical condition values and could be specially applied during confinement, when conducting epidemiological studies, or as screening tests in particular situations where time is money.

## Figures and Tables

**Table 1:** Participants’ anthropometry and physical fitness tests stratified by sex.

	**Boys**	**Girls**	
**N (%)**	108 (48)	117 (52)	
	**Median (IR)**	**Median (IR)**	***p***
**Age (years)**	13.00 (1.00)	13.00 (1.00)	0.758
**Weight (kg)**	52.90 (20.95)	49.80 (11.05)	0.130
**BMI (kg/m^2^)**	19.8 (5.4)	20.40 (3.85)	0.314
**FM (kg)**	9.30 (7.65)	13.5 (5.5)	<0.001 *
**FFM (kg)**	42.35 (16.1)	36.60 (7.15)	<0.001 *
**HS (kg)**	29.15 (11.77)	23.4 (6.95)	<0.001 *
**CF (stage)**	6.25 (3.87)	4.00 (2.5)	<0.001 *
**SA (sec)**	11.36 (1.95)	12.20 (1.95)	<0.001 *
	**Mean (SD)**	**Mean (SD)**	***p***
**Height (cm)**	161.80 (10.71)	158.02 (7.03)	0.002 ^†^
**FM% (%)**	18.35 (9.55)	26.1 (6.55)	<0.001 ^†^
**SLJ (cm)**	168.5 (43)	141 (30.5)	<0.001 ^†^

FM: fat mass; FFM; fat-free mass; HS: handgrip strength; CF: cardiorespiratory fitness; SA: speed–agility; FM%: fat mass percent; SLJ: standing long jump. Data are shown as mean (standard deviation) and median (interquartile range). * Significant differences for *p* ≤ 0.05 in nonparametric variables; ^†^ Significant differences for *p* ≤ 0.05 in parametric variables.

**Table 2:** Participants’ anthropometry and physical fitness tests stratified by BMI categories.

	**Low Weight (a)**		**Normal Weight (b)**		**Overweight and Obese (c)**		
**N (%)**	5 (2.2)		87 (38.7)		133 (59.1)		
	Boys (1.7)	Girls (0.4)	Boys (19.1)	Girls (19.5)	Boys (27.1)	Girls (32.0)	
	**Median (IR)**		**Median (IR)**		**Median (IR)**		***p***
**Weight (kg)**	33.10 (8.65)		45.80 (12.20)		55.10 (14.80)		
**BMI (kg/m2)**	14.8 (0.85)		17.50 (2.20)		22.00 (3.25)		<0.001 (ab, ac, bc) *
**FM (kg)**	3.70 (3.00)		8.10 (3.80)		14.70 (5.70)		0.003 (ab) * <0.001 (ac, bc) *
**FFM (kg)**	28.30 (6.60)		36.80 (10.40)		41.20 (11.30)		0.008 (ab) * <0.001 (ac, bc) *
**HS (kg)**	24.10 (10.60)		22.80 (8.70)		26.5 (9.00)		0.014 (bc) *
**CF (stage)**	6.00 (2.75)		6.00 (4.00)		4.50 (3.00)		<0.001 (bc) *
**SA (sec)**	11.76 (1.25)		11.40 (1.80)		12.00 (2.33)		0.002 (bc) *
	**Mean (SD)**		**Mean (SD)**		**Mean (SD)**		***p***
**Height (cm)**	148.60 (9.98)		159.94 (8.80)		160.19 (9.17)		
**FM%**	14.30 (3.58)		19.17 (4.69)		26.90 (5.56)		0.035 (ab) ^†^ 0.001 (ac) ^†^ <0.001 (bc) ^†^
**SLJ (cm)**	160.00 (21.71)		160.59 (31.29)		148.73 (26.44)		0.004 (bc) ^†^

(a) Low weight: BMI < −3 SD; (b) normal weight: BMI < −2 SD; (c) overweight and obese: BMI > +1 SD. FM: fat mass; FFM; fat-free mass; HS: handgrip strength; CF: cardiorespiratory fitness; SA: speed–agility; FM%: fat mass percent; SLJ: standing long jump. Data are shown as mean (standard deviation) and median (interquartile range). * Significant differences for *p* ≤ 0.05 in nonparametric variables; ^†^ Significant differences for *p* ≤ 0.05 in parametric variables.

**Table 3:** Obese and overweight adolescents’ anthropometry and physical fitness tests stratified by sex.

	**Boys**	**Girls**	
**N (%)**	61 (45.9)	72 (54.1)	
	**Median (IR)**	**Median (IR)**	***p***
**Weight (kg)**	61.50 (17.10)	53.25 (12.27)	0.002 *
**BMI (kg/m2)**	22.10 (3.25)	21.90 (3.50)	0.385
**FM (kg)**	13.90 (5.85)	15.30 (4.95)	0.020 *
**FFM (kg)**	45.90 (13.35)	37.90 (7.23)	<0.001 *
**HS (kg)**	30.50 (10.70)	24.20 (8.25)	<0.001 *
**CF (stage)**	5.00 (3.50)	3.75 (2.00)	<0.001 *
**SA (sec)**	11.50 (2.53)	12.45 (2.12)	<0.001 *
	**Mean (SD)**	**Mean (SD)**	***p***
**Height (cm)**	163.18 (10.14)	157.6 (7.44)	0.001 ^†^
**FM%**	23.90 (5.01)	29.45 (4.67)	<0.001 ^†^
**SLJ (cm)**	158.54 (29.10)	140.42 (20.78)	<0.001 ^†^

FM: fat mass; FFM; fat-free mass; HS: handgrip strength; CF: cardiorespiratory fitness; SA: speed–agility; FM%: fat mass percent; SLJ: standing long jump. Data are shown as mean (standard deviation) and median (interquartile range). * Significant differences for *p* ≤ 0.05 in nonparametric variables; ^†^ Significant differences for *p* ≤ 0.05 in parametric variables.

**Table 4:** Bivariate correlations between predictors and outcome variables stratified by sex and BMI range.

	BMI (kg/m^2^)	FM%	FM (kg)	FFM (kg)	SLJ (cm)	SA (sec)	CF (stage)
**All participants**							
FM%	0.682 **						
FM (kg)	0.894 **	0.882 **					
FFM (kg)	0.626 **	0.017	0.448 **				
SLJ (cm)	−0.148	−0.526 **	−0.342 **	0.272 **			
SA (sec)	0.093	0.406 **	0.232 **	−0.287 **	−0.527 **		
CF (Stage)	−0.233 **	−0.563 **	−0.421 **	0.162	0.583 **	−0.563 **	
HS (kg)	0.334 **	−0.118	0.185 **	0.637 **	0.375 **	−0.312 **	0.298 **
**Boys**							
FM%	0.748 **						
FM (kg)	0.945 **	0.868 **					
FFM (kg)	0.724 **	0.194	0.631 **				
SLJ (cm)	−0.151	−0.509 **	−0.276 **	0.196			
SA (sec)	0.056	0.382 **	0.178	−0.262 **	−0.605 **		
CF (Stage)	−0.235	−0.545 **	−0.353 **	0.137	0.627 **	−0.529 **	
HS (kg)	0.420 **	−0.055	0.286 **	0.670 **	0.446 **	−0.417 **	0.374 **
**Girls**							
FM%	0.811 **						
FM (kg)	0.907 **	0.907 **					
FFM (kg)	0.658 **	0.350 **	0.674 **				
SLJ (cm)	−0.057	−0.219	−0.109	0.171			
SA (sec)	0.050	0.183	0.050	−0.251 **	−0.337 **		
CF (Stage)	−0.184	−0.222	−0.216	−0.073	0.321 **	−0.486 **	
HS (kg)	0.336 **	0.243 *	0.416 **	0.502 **	0.175	−0.114	0.026
**Normal-weight adolescents**							
FM%	0.253 **						
FM (kg)	0.670 **	0.804 **					
FFM (kg)	0.432 **	−0.383 **	0.094				
SLJ (cm)	0.165	−0.571 **	−0.301 **	0.478 **			
SA (sec)	−0.338 **	0.268 *	−0.013	−0.439 **	−0.497 **		
CF (Stage)	0.096	−0.604 **	−0.389 **	0.350 **	0.662 **	−0.519 **	
HS (kg)	0.438 **	−0.373 **	0.033	0.702 **	0.608 **	−0.455 **	0.445 **
**Obese and overweight adolescents**							
FM%	0.439 **						
FM (kg)	0.791 **	0.772 **					
FFM (kg)	0.568 **	−0.277 **	0.339 **				
SLJ (cm)	−0.082	−0.499 **	−0.306 **	0.289 **			
SA (sec)	0.038	0.469 **	0.248 *	−0.348 **	−0.543 **		
CF (Stage)	−0.090	−0.493 **	−0.309 **	0.236 **	0.523 **	−0.590 **	
HS (kg)	0.302 **	−0.223 *	0.148	0.594 **	0.292 **	−0.292 **	0.311 **

FM%: fat mass percent; FM: fat mass; FFM; fat-free mass; SLJ: standing long jump; SA: speed–agility; CF: cardiorespiratory fitness; HS: handgrip strength. * Significant correlation at level 0.01. ** Significant correlation at level 0.007.
